# Economic evaluation of operative versus nonoperative treatment of a humeral shaft fracture: economic analyses alongside a multicenter prospective cohort study (HUMMER)

**DOI:** 10.1007/s00068-022-02160-1

**Published:** 2022-12-08

**Authors:** Saskia H. Van Bergen, Esther M. M. Van Lieshout, Kiran C. Mahabier, Alexandra J. L. M. Geraerds, Suzanne Polinder, Dennis Den Hartog, Michael H. J. Verhofstad, the HUMMER Investigators, Ivo Beetz, Hugo W. Bolhuis, P. Koen Bos, Maarten W. G. A. Bronkhorst, Milko M. M. Bruijninckx, Jeroen De Haan, Axel R. Deenik, P. Ted Den Hoed, Martin G. Eversdijk, J. Carel Goslings, Robert Haverlag, Martin J. Heetveld, Albertus J. H. Kerver, Karel A. Kolkman, Peter A. Leenhouts, Sven A. G. Meylaerts, Ron Onstenk, Martijn Poeze, Rudolf W. Poolman, Bas J. Punt, Ewan D. Ritchie, W. Herbert Roerdink, Gert R. Roukema, Jan Bernard Sintenie, Nicolaj M. R. Soesman, Edgar J. T. Ten Holder, Wim E. Tuinebreijer, Maarten Van der Elst, Frank H. W. M. Van der Heijden, Frits M. Van der Linden, Peer Van der Zwaal, Jan P. Van Dijk, Hans-Peter W. Van Jonbergen, Egbert J. M. M. Verleisdonk, Jos P. A. M. Vroemen, Marco Waleboer, Philippe Wittich, Wietse P. Zuidema, Ahmed Al Khanim, Jelle E. Bousema, Kevin Cheng, Yordy Claes, J. Daniël Cnossen, Emmelie N. Dekker, Aron J. M. De Zwart, Priscilla A. Jawahier, Boudijn S. H. Joling, Cornelia A. W. Notenboom, Jaap B. Schulte, Nina Theyskens, Gijs J. J. Van Aert, Boyd C. P. Van der Schaaf, Tim Van der Torre, Joyce Van Veldhuizen, Lois M. M. Verhagen, Maarten Verwer, Joris Vollbrandt

**Affiliations:** 1 Trauma Research Unit, Department of Surgery, Erasmus MC, University Medical Center Rotterdam, Rotterdam, The Netherlands; 2 Department of Public Health, Erasmus MC, University Medical Center Rotterdam, Rotterdam, The Netherlands

**Keywords:** Cost-effectiveness, Cost-utility, Fracture, Health care consumption, Humerus, Nonoperative, Operative, Shaft

## Abstract

**Purpose:**

Operative treatment of a humeral shaft fracture results in faster recovery than nonoperative treatment. The cost-effectiveness, in terms of costs per Quality-Adjusted Life Year (QALY) gained (Dutch threshold €20,000-€80,000) or minimal important change (MIC) in disability reduced (DASH 6.7), is unknown. The aim of this study was to determine cost-utility and cost-effectiveness of operative versus nonoperative treatment in adults with a humeral shaft fracture type 12A or 12B.

**Methods:**

This study was performed alongside a multicenter prospective cohort study. Costs for health care and lost productivity until one year after trauma were calculated. The incremental cost-utility ratio (ICUR) was reported in costs per QALY (based on the EuroQoL-5D-3L (EQ-5D)) gained. The incremental cost-effectiveness ratio (ICER) was reported in costs per MIC (based on the DASH score at three months) reduced.

**Results:**

Overall, 245 patients were treated operatively and 145 nonoperatively. In the operative group, the mean total costs per patient (€11,925 versus €8793; *p* < 0.001) and QALYs (0.806 versus 0.778; *p* < 0.001) were higher. The ICUR of operative treatment was €111,860 per QALY gained (*i.e.*, €3132/0.028). The DASH was 7.3 points (*p* < 0.001) lower in the operative group. The ICER of operative treatment was €2880 per MIC in disability reduced (*i.e.*, €3132/7.3*6.7).

**Conclusion:**

Due to the limited effect of treatment on quality of life measured with the EQ-5D, the ICUR of operative treatment (€111,860 per QALY gained) exceeds the threshold. However, the incremental costs of €2880 per clinically meaningful difference in DASH are much lower and suggest that operative treatment for a humeral shaft fracture is cost-effective.

**Supplementary Information:**

The online version contains supplementary material available at 10.1007/s00068-022-02160-1.

## Background

In an era of budget restraints on health care costs, efficient resource use is crucial and data on cost-effectiveness of treatment are gaining importance in health care budget allocation [1–3]. In the Netherlands, costs of injuries account for 5% of the total health care budget and 8% of the indirect costs resulting from all diseases [3]. However, there seems to be a paucity of evidence in the area of cost-utility and cost-effectiveness of treatment of orthopedic trauma injuries [2]. Multiple studies have shown that long bone fractures are costly in terms of direct medical costs and lost productivity [4, 5]. The burden on society of long bone fractures can be attributed to the costs of surgery, possible reinterventions, and the physical rehabilitation of patients [5]. When comparing upper extremity injuries, upper arm fractures resulted in the highest costs per case (€4440) in the Netherlands [6]. Cumulative medical costs in the Netherlands of patients, admitted due to a humeral shaft fracture only, added up to €10.6 million in 2012 [7].

Humeral shaft fractures pose a burden on society as they make up 3% of all orthopedic injuries [8]. In the Netherlands, the overall incidence rate of patients admitted for a humeral fracture per year has risen by 132% to 7.2 per 100,000 person years from 1986 to 2012, partly attributable to an aging population [7]. Incidence rate is characterized by a bimodal age distribution, affecting both young and elderly patients, which influences the pattern of health care costs [7, 9]. Fractures in young employed persons can induce high costs due to the absence of work and lost productivity [4]. Furthermore, it is established that especially medical costs of humeral shaft fractures in elderly women are substantial due to extended nursing home admission or homecare [7, 9].

Humeral shaft fractures can be managed operatively or nonoperatively, with both treatments resulting in high union rates and excellent results [8]. Nonoperative treatment is mostly performed using a functional brace [10]. Operative treatment mostly includes plate osteosynthesis, intramedullary nailing (IMN), or external fixation for limited indications [8]. The primary results of the HUMMER study indicate, based on functional and clinical outcomes, that operative treatment should be the preferred treatment option for these fractures, as it is associated with faster functional recovery and fewer complications such as nonunion than nonoperative treatment [11].

These findings are not yet supported by data on health care consumption and costs [12]. Policy-makers need the detailed information provided by cost-utility (CUA) and cost-effectiveness analyses (CEA) to adequately balance costs and effects with suitable thresholds of efficiency in order to provide well-informed advice on health care budget allocation [9, 13–15]. Therefore, the aim of this study was to determine cost-utility and cost-effectiveness of operative versus nonoperative treatment of adult patients with a humeral shaft fracture. The hypothesized was that operative treatment would be cost-effective, due to earlier functional recovery and lower costs for follow-up and lost productivity outweighing higher costs for initial treatment.

## Methods

### Setting and participants

These economic analyses were performed alongside the observational HUMMER study [16]. The study was exempted by the local Medical Research Ethics Committee (no. MEC-2012–296) and registered at the Netherlands Trial Register (NTR3617). Patients were eligible if they (1) were aged 18 years and older (with no upper limit), (2) had a closed fracture of the humeral shaft (AO type 12A or 12B; confirmed on X-ray), (3) had provided written informed consent, and if operatively treated, (4) had an operation within 14 days after presentation to the Emergency Department [17]. Patients were excluded if they had sustained other traumatic injuries or were known to have pre-existing disorders that were expected to affect bone healing, treatment, or rehabilitation of the affected arm (*e.g.*, polytrauma, open fractures, pathological fractures, bone disorders (excluding osteoporosis), rheumatoid arthritis, or pre-existing impaired upper extremity function). Furthermore, patients with expected problems with follow-up (*e.g.,* no fixed address or cognitive impairment) or insufficient comprehension of the Dutch language were excluded. Full details on inclusion and exclusion criteria are available in the published study protocol [16].

Treatment was left to the treating physician and consisted either of operative treatment with plate osteosynthesis or IMN, or nonoperative treatment with a splint, plaster, collar and cuff, or hanging cast, followed by a Sarmiento brace.

### Outcomes measures

The effect measure for the CUA was the Quality-Adjusted Life Years (QALYs). The mean increase in QALYs during one year was calculated using the EuroQol-5D-3L (EQ-5D), a validated questionnaire recommended for assessing quality of life in trauma patients, especially for economic assessments [18–20]. Participants completed the EQ-5D at two and six weeks and three, six, and 12 months after initiation of treatment. The EQ-5D descriptive system consists of five health domains (mobility, self-care, usual activities, pain/discomfort, and anxiety/depression) with three answer levels (no problem, moderate problem, or severe problem). Utility scores were calculated to express the health status descriptions ranging from zero to one, in which zero is death and one is full health.

The effect measure for the CEA was the Disabilities of the Arm, Shoulder, and Hand (DASH) score at three months, as at that time, a clinical difference was expected [21]. The DASH is a validated, 30-item (scored 1–5), self-report questionnaire with an overall score ranging from 0 (no disability) to 100 (severe disability), reflecting functional outcome and pain of the upper extremity [22, 23]. The minimal important change (MIC) of the DASH is 6.7 points [21].

### Health care consumption and productivity loss measurement

These economic analyses were performed from a societal perspective, following Dutch guidelines [24, 25]. Data on health care consumption and work absenteeism were collected at each scheduled follow-up contact using a custom-made questionnaire based upon the Medical Consumption Questionnaire (iMCQ) and the iMTA Productivity Cost Questionnaire (iPCQ) [26, 27]. Data were gathered until one year after trauma. Health care consumption included intramural and extramural medical care directly associated with diagnosis, treatment, and rehabilitation of the patient with a humeral shaft fracture. Missing data of hospital care consumption were collected during the close-out visits at each hospital.

### Cost calculation

Reference prices of health care resources were derived from the Dutch manual for costing in economic evaluations where possible (Supplemental Table S1-2) [28]. Other reference prices for cost categories were calculated based on data derived from the participating academic and non-academic hospitals, surgical equipment and implant firms, the NZa (Nederlandse Zorgautoriteit; Dutch Healthcare Authority), the CVZ (College voor Zorgverzekeringen; Health Care Insurance Board), or obtained from home care firms [24, 29]. Reference unit costs for 2020 (€) were used or adjusted to 2020 (€) costs with the national consumer price index [30]. Inflation was taken into account. Costs were calculated by multiplying the frequency of resource use by the unit prices per cost category. Comparison with US costs was done after applying the exchange rate (€1 = US$1.21) [31].

Indirect societal costs due to work absence were calculated using the friction cost method [18]. Costs for lost productivity were defined as the costs associated with production loss and replacement due to illness, disability, and premature death [32]. Costs for lost productivity were calculated by multiplying the cumulative duration of work absence in hours within the first 85 days after injury with the costs related to work absenteeism for different five-year age groups for employed persons aged 18–68 years (Supplemental Table S1) [33].

### Statistical analysis

Data were analyzed using the Statistical Package for the Social Sciences version 25 (SPSS, Chicago, Ill., USA). Missing data were not imputed. Data were averaged for patients for whom data were available. Analysis was performed according to intention to treat and all statistical tests were two-sided. Chi-squared analysis was used for statistical testing of categorical data. Functional outcomes that were repeatedly measured over time were compared between treatment groups using linear mixed-effects regression models, as described before [11]. The models included fixed effects for treatment group, age, gender, and the individual fracture types. Continuous data were analyzed using a Mann–Whitney *U* test. For the pairwise comparison of the mean costs, the bootstrap 95% confidence interval (95% CI) was computed based on 1000 replications. Since the time horizon was one year, no discounting was required for costs and health utilities. Results were reported following the CHEERS Checklist for reporting economic health evaluations [34]. A *p* value < 0.05 was taken as a threshold for statistical significance in all statistical tests.

The incremental cost-utility ratio (ICUR), comparing operative versus nonoperative treatment, was expressed in terms of incremental mean total costs per mean QALY gained and calculated by dividing the difference of the mean total costs by the difference of the mean increase in QALYs over 12 months. The Dutch threshold of maximum costs per QALY was used (ranging from €20,000 up to €80,000 per QALY) [9, 13–15, 35].

The incremental cost-effectiveness ratio (ICER), comparing operative treatment versus nonoperative treatment, was reported in terms of incremental costs for a clinically meaningful difference (6.7 DASH points reduced at the three months’ time point). The ICER was calculated by dividing the difference of the mean total costs of the two interventions by the difference of the mean DASH score at three months and multiplied by 6.7. This ratio, with a different time interval used in the numerator and denominator, was chosen in order to compare clinical expected differences to the total costs of treatment, as a difference in DASH score was expected at three months and treatment of a humeral shaft fracture usually does not exceed one year [16].

## Results

### Patient characteristics and employment details

Between October 23, 2012 and October 3, 2018, 390 patients were included of whom 245 (62.8%) were treated operatively and 145 (37.2%) nonoperatively. Compared with the nonoperative group, patients in the operative group were younger (median age of 53 (P_25_–P_75_ 35–66) versus 62 (P_25_–P_75_ 49–71) years; *p* < 0.001) and more often male (45.6% versus 35.2%; *p* = 0.044) (Table 1). Furthermore, patients in the operative group were significantly more often employed (55.5% versus 42.8% in the nonoperative group; *p* = 0.016) and worked more hours per week (38 versus 32 h in the nonoperative group; *p* = 0.016). Twenty patients were lost to follow-up due to mortality (*N* = 4) or withdrawal of consent (*N* = 16). The total number of patients available for follow-up varied per follow-up moment, as 55 patients did not show up at least one follow-up visit.

**Table 1:** Patient characteristics and employment details

	All (*N* = 390)		Operative (*N* = 245)		Nonoperative (*N* = 145)		*P* value
	N*		N*		N*		
Patient characteristics							
Female	390	227 (58.2%)	245	133 (54.3%)	145	94 (64.8%)	**0.044**
Age (year)	390	57 (40–68)	245	53 (35–66)	145	62 (49–71)	** < 0.001**
Work							
Employed	390	198 (50.8%)	245	136 (55.5%)	145	62 (42.8%)	**0.016**
Hours per week	194	36 (27–40)	134	38 (32–40)	60	32 (21–40)	**0.016**

*P* values < 0.05 are shown in boldface

Data are presented as *N* (%) or median (P_25_–P_75_)

N* represents the number of patients for whom data were available per follow-up moment

### QALY and DASH

The mean increase in QALYs during one year was 0.028 higher after operative treatment (mean of 0.806 (95% CI 0.801–0.811) versus 0.778 (95% CI 0.771–0.784) in the nonoperative group; *p* < 0.001), which was mostly attributable to a faster increase in health-related quality of life in the first six months.

There was a significant and clinically meaningful difference in DASH score of 7.3 points between the operative and nonoperative group at three months follow-up, in favor of the operative group (mean of 22.3 (95% CI 19.9–24.6) versus 29.6 (95% CI 26.6–32.6) in the nonoperative group; *p* = 0.001).

### Health care consumption and work absence.

An overview of the mean health care consumption and work absenteeism per patient is shown in Table 2. Patients in the operative group were all admitted to the hospital (*N* = 145, 100%) for a median stay of 2 (P_25_–P_75_ 2–4) days. In the nonoperative group, 26 (17.9%) patients were admitted for a median stay of 2 (P_25_–P_75_ 2–3) days. Patients in the operative group had significantly more medical imaging units during their primary stay compared to the nonoperative group (median 4 (P_25_–P_75_ 2–4) versus 2 (P_25_–P_75_ 2–2) units; *p* < 0.001). During follow-up, patients in the nonoperative group had significantly more medical imaging, used more devices for immobilization, and had more outpatient clinic visits. Besides that, compared with the operative group, a doubling of surgical reinterventions was found in the nonoperative group (12.2% (*N* = 30) versus 25.5% (*N* = 37); *p* < 0.001). Reinterventions in the operative group (*N* = 30) were performed due to implant-related complications (*N* = 19), nonunion (*N* = 10), and a deep infection (*N* = 1). Surgical interventions in the nonoperative group (*N* = 37) consisted of conversions to osteosynthesis of the humeral shaft fracture due to nonunion (*N* = 20), malunion (*N* = 11), pain (*N* = 5), and persistent radial nerve apraxia (*N* = 2).

**Table 2:** Mean health care consumption and work absenteeism by treatment group

		All (*N* = 390)		Operative (*N* = 245)		Nonoperative (*N* = 145)		*P* value
		N*		N*		N*		
**Health care consumption - primary stay**								
Ambulance transport	Rides	390	1 (0–1)	245	1 (1–1)	145	1 (1–1)	1.000
Emergency department	Visits	390	1 (1–1)	245	1 (1–1)	145	1 (1–1)	1.000
Medical imaging	Units	390	2 (2-4)	245	4 (2-4)	145	22 (2-2)	**< 0.001**
Initial treatment								
Operation time (including anesthesia)	Minutes	194	120 (96–152)	194	120 (96–152)	N.A	N.A	N.A
Operation time (in theater)	Minutes	224	81 (65–112)	224	81 (65–112)	N.A	N.A	N.A
Immobilization	Units	390	1 (1–1)	244	1 (1–1)	145	1 (1–2)	1.000
Admission and follow-up characteristics								
Hospital	Admission	390	271 (69.5%)	245	245 (100.0%)	145	26 (17.9%)	** < 0.001**
	LOS (days)	271	2 (2–4)	245	2 (2–4)	26	2 (2–3)	0.830
**Health care consumption - follow-up**								
Medical imaging	Units	390	11 (8–14)	245	10 (8–12)	145	12 (10–15)	** < 0.001**
Immobilization	Units	390	0 (0–0)	245	0 (0–0)	145	0 (0–0)	** < 0.001**
Outpatient clinic	Visits	390	5 (3–6)	390	4 (3–6)	390	5 (4–7)	** < 0.001**
General practitioner	Visits	318	0 (0–1)	201	0 (0–1)	117	0 (0–1)	0.341
Emergency department	Visits	318	0 (0–0)	201	0 (0–0)	117	0 (0–0)	0.970
Adverse events								
Any surgical reintervention	Number	390	67 (17.2%)	245	30 (12.2%)	145	37 (25.5%)	**0.001**
Operation time (including anesthesia)	Minutes	56	86 (43–130)	23	50 (27–99)	33	93 (69–153)	0.103
Operation time (in theater)	Minutes	64	118 (77–172)	27	86 (52–162)	37	125 (102–192)	0.311
Hospital admission	LOS (days)	46	1 (1–3)	12	2 (1–4)	34	1 (1–3)	0.988
**Discharge disposition resulting in changes in living situation**								
Nursing home	LOS (days)	1	30 (30–30)	1	30 (30–30)	0	N.A	N.A
Care hotel	LOS (days)	7	10 (5–30)	4	8 (5–25)	3	21 (3–21)	0.721
Elderly care facility	LOS (days)	4	35 (23–84)	1	21 (21–21)	3	42 (28–42)	0.180
Rehabilitation clinic	LOS (days)	3	25 (24–25)	3	25 (24–25)	0	N.A	N.A
**Health care consumption related to rehabilitation**								
Physical therapy	Number of sessions	343	22 (10–45)	217	25 (12–48)	125	20 (10–40)	0.392
Home care	Hours	318	0 (0–0)	201	0 (0–0)	117	0 (0–0)	0.506
Other rehabilitation therapy	Number of sessions	318	0 (0–0)	201	0 (0–0)	117	0 (0–0)	0.084
**Work**								
Work absence	% of employed patients	196	179 (91.3%)	134	123 (91.8%)	62	56 (90.3%)	0.787
Work days missed	Days	196	30 (13-54)	134	26 (12-49)	62	33 (15-59)	0.253

*P* values < 0.05 are shown in boldface

Data are presented as *N* (%) or median (P_25_–P_75_)

N* represents the number of patients for whom data were available per follow-up moment

LOS, Length of Stay

Although the operative group resumed work seven days earlier (26 versus 33 days in the nonoperative group), there was no significant difference in work absence in days (*p* = 0.253).

### Health care costs and costs for lost productivity

An overview of the mean health care costs per patient is shown in Table 3. The mean total costs were significantly higher in the operative group (€11,925 versus €8793 in the nonoperative group; *p* < 0.001) (Table 3). In addition, the mean total hospital costs per patient of primary stay were significantly higher in the operative group (€5159 versus €1093; *p* < 0.001). The mean costs of surgery attributed to almost half of the costs of primary stay (€2434). The mean follow-up costs per person were significantly lower in the operative group (€1377 versus €2306; *p* < 0.001). The mean costs for ambulance transport, medical imaging (primary stay), initial treatment, and hospital admission days (primary stay) were significantly higher in the operative group. The mean costs of devices for immobilization (initial treatment), medical imaging (follow-up), and mean costs related to revision surgery and consequent hospital admission days were significantly lower in the operative group.

**Table 3:** The mean costs (2020) (€) by treatment group

	All (*N* = 390)		Operative (*N* = 245)		Nonoperative (*N* = 145)		Mean difference in costs	*P* value
	N*		N*		N*			
**Hospital costs - primary stay**								
Ambulance transport	390	391 (355–427)	245	435 (387–480)	145	317 (256–377)	118 (23–201)	**0.018**
Emergency department visit	390	280 (280–280)	245	280 (280–280)	145	280 (280–280)	0 (0–0)	–
Medical imaging	390	211 (200–222)	245	244 (231–257)	145	155 (139–174)	89 (71–115)	**0.001**
Initial treatment								
Surgical costs	335	1380 (1234–1520)	190	2434 (2337–2532)	N.A	N.A	2434 (2265–2526)	**0.001**
Immobilization	390	41 (36–46)	245	12 (10–15)	145	90 (82–98)	− 78 (− 85 to − 69)	**0.001**
Hospital admission days	390	1188 (1041–1330)	245	1742 (1566–1935)	145	251 (154–350)	1491 (1336–1677)	**0.001**
Total hospital costs – primary stay	335	3399 (3130–3672)	190	5159 (4901–5441)	145	1093 (974–1219)	4066 (3577–4268)	**0.001**
**Hospital costs – follow-up**								
Medical imaging	390	683 (659–708)	245	636 (609–668)	145	761 (720–799)	− 125 (− 163 to –55)	**0.001**
Outpatient clinic visits	390	419 (387–454)	245	396 (356–445)	145	458 (420–494)	− 62 (− 130 to 10)	0.095
General practitioner visits	318	18 (15–21)	201	18 (14–23)	117	16 (11–22)	2 (− 5 to 10)	0.462
Emergency department visits	318	11 (5–17)	201	10 (3–18)	117	12 (2–24)	− 2 (− 16 to 12)	0.825
Medication	390	84 (66–102)	245	82 (60–107)	145	89 (61–124)	− 7 (− 67 to 20)	0.302
Immobilization	390	4 (2–6)	245	2 (1–4)	145	6 (3–11)	− 4 (− 5 to 1)	0.192
Adverse events								
Revision surgery	378	363 (265–470)	238	159 (91–237)	140	708 (508–93)	− 549 (− 742 to − 268)	**0.001**
Hospital admission days	390	124 (85–165)	245	61 (20–114)	145	229 (145–330)	− 168 (− 260 to − 35)	**0.020**
Total hospital costs – follow-up	306	1717 (1548–1900)	194	1377 (1229–1551)	112	2306 (1935–2685)	− 929 (− 1250 to − 444)	**0.001**
**Costs related to rehabilitation/changes in living situation**								
Discharge disposition	318	501 (220–855)	201	553 (171–1074)	117	413 (83–864)	140 (− 289 to 1175)	0.203
Home care	318	836 (505–1201)	201	593 (303–970)	117	1,250 (568–2123)	− 657 (− 1584 to 13)	0.099
Rehabilitation therapy								
Physical therapy	343	1087 (971–1199)	217	1148 (1014–1288)	125	981 (828–1143)	167 (− 63 to 424)	0.134
Other rehabilitation therapy	318	18 (7–32)	201	21 (8–38)	117	14 (0–42)	7 (− 21 to 42)	0.563
Total costs related to rehabilitation/ changes in living situation	318	2473 (1942–3023)	201	2324 (1765–2982)	117	2731 (1913–3736)	− 407 (− 1388 to 1085)	0.818
**Indirect costs**								
Costs for lost productivity	318	2894 (2471–3338)	201	3007 (2449–3623)	117	2702 (1986–3422)	305 (− 849 to 1266)	0.692
**Total costs**	263	10,615 (9681–11,531)	153	11,925 (10,791–13,153)	110	8793 (7584–10,140)	3132 (1325–4940)	**0.001**

The exchange rate was €1 = US$1.21 [31]

*P* values < 0.05 are shown in boldface

Data are presented as a mean with a bootstrap 95% CI

N* represents the number of patients for whom data were available per follow-up moment

**Fig. 1:**
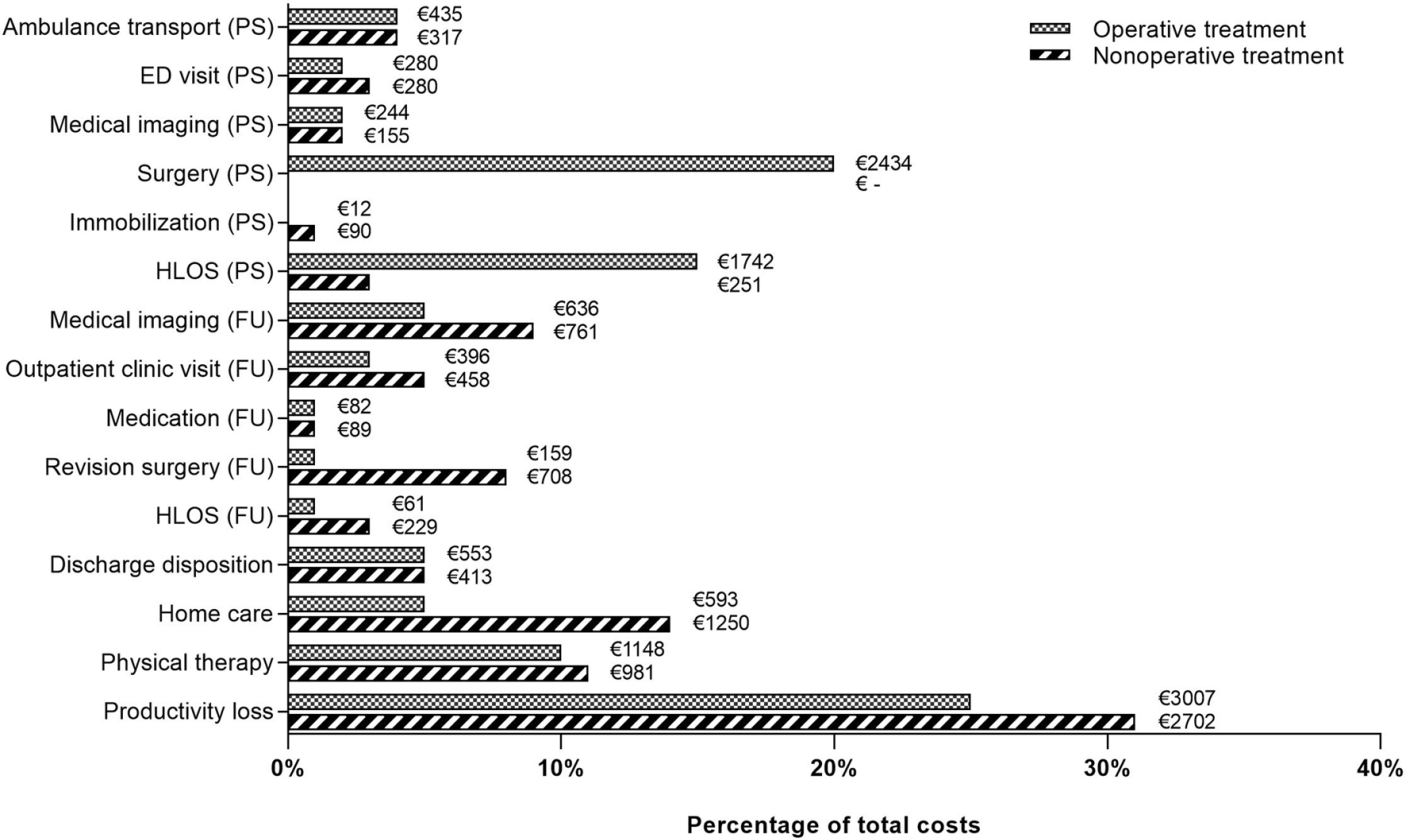
The relative contribution of various cost categories. The exchange rate was €1.00 = US$1.21 [31]. Only cost categories representing more than 1% of the total costs are shown. *ED* Emergency department, *FU* Follow-up, *HLOS* Hospital length of stay, *PS* Primary stay

The main cost drivers for operative treatment were costs for lost productivity (25%), surgery (20%), hospital admission (primary stay) (15%), and physical therapy (10%) (Table 3 and Fig. 1). The main cost drivers for nonoperative treatment were costs for lost productivity (31%), home care (14%), physical therapy (11%), and revision surgery (8%).

### Cost-utility analysis

Operative treatment resulted in higher mean total costs per person until 12 months of €3132 (95% CI €1325–€4940; *p* < 0.001). The mean change in QALYs until 12 months was 0.028 (*p* < 0.001) higher in the operative group. Hence, this resulted in incremental costs for operative treatment of €111,857 (*i.e.*, €3132/0.028) per QALY gained.

### Cost-effectiveness analysis

The mean difference in DASH score was 7.3 points (*p* < 0.001) in favor of operative treatment, resulting in incremental costs for operative treatment of €2880 (*i.e.*, €3132/7.3*6.7) for a meaningful change in disability.

## Discussion

This study showed that operative treatment of a humeral shaft fracture results in higher mean costs per person over one year of €3132 (95% CI €1325–€4940; *p* < 0.001) than nonoperative treatment. The mean difference in QALYs (0.028; *p* < 0.001) during one year in favor of the operative group demonstrates that operative treatment results in a higher health-related quality of life during the first year after trauma. This difference is statistically significant but small, therefore incremental costs per QALY gained are high (€111,857; *i.e.*, €3132/0.028). The clinical and statistically significant difference of 7.3 DASH points (*p* < 0.001) in favor of the operative group exceeds the MIC and results in incremental costs for operative treatment of €2880 for a measurable change in disability.

The different measures of efficacy used in these economic analyses should be carefully weighted in the decision-making process. Economic evaluations with QALYs may be preferred in order to allow for comparison across populations with different medical conditions. However, a humeral shaft fracture does not necessarily affect a patient’s self-reported health-related quality of life as the injury may have little effect on some of the measured domains of the EQ-5D (*i.e.,* anxiety and depression), resulting in marginal differences in QALYs gained [21]. Due to the limited effect of a humeral shaft fracture on quality of life, the costs per QALY (€111,857) exceed the threshold set by society. The difference in functional outcome measured by the DASH score was shown to be more specific than the health-related quality of life measured in QALYs [21]. An ICER calculated with the DASH score cannot be compared to other injuries, but it does show the relatively low incremental costs of operative treatment for a clinically meaningful difference and suggests that operative treatment for a humeral shaft fracture is cost-effective.

The results of the cost calculations are comparable with results from previous research. Polinder *et* *al*. (2013) described comparable direct health care costs of upper arm fractures of €4440 per case (versus €5116 in this study), taking into account inflation and the more detailed health care resource use described in this study [6]. Bonafede* et* *al*. (2013) determined higher direct health care costs (US$10,842 (≈ €8960) versus €7589) and higher costs for lost productivity (US$4868 (≈ €4023 versus €2894 in this study) per humeral fracture [4]. However, costs were calculated by multiplying the total number of hours reported absent multiplied by an average rate per hour (human capital approach) instead of assuming that productivity costs are only incurred during the period until the moment the employee is replaced, the so-called friction period [4, 32]. Meerding *et* *al*. (2006) described similar total costs of humeral shaft fractures in the Netherlands, namely €9430 per patient, with also hospital care costs and costs for lost productivity as main cost drivers [36].

Patients’ preferences shape clinical decision-making which therefore could be influenced by employment status. It is desirable that employed patients return to work as soon as possible, especially knowing that costs for lost productivity account for more than a quarter of the total costs of treatment of a humeral shaft fracture and added up to €5.4 million in the Netherlands for admitted patients alone in 2012 [7]. Hendy *et* *al. *(2020) identified no advantage for faster return to work after operative or nonoperative treatment of humeral shaft fractures [37]. This study showed that employed patients were treated operatively more often, but there was no significant difference in work absence in days or costs for lost productivity between treatment groups. However, the underlying differences between the treatment groups, specifically the male predominance, younger median age, and overrepresentation of employed patients, who also worked significantly more hours per week, in the operative treatment group, result in an underestimation of the advantage of their earlier return to work in terms of costs for lost productivity.

## Strengths and limitations

The strengths of this study include a large multicenter prospective cohort methodology measuring health utility, a formal economic costing approach, and a societal perspective for costs. Furthermore, this study design ensures great external validity by allowing for variation between hospitals (*e.g.*, differing policies on follow-up procedures and allocation of resources).

A limitation of these cost analyses is that both groups included multiple treatment strategies with different costs of material (Supplemental Table S1). Moreover, costs were based on Dutch prices and practices and therefore may vary depending on the health care system used. Furthermore, the follow-up duration of 12 months did not take into account the late complications of nonunion or the need for revision surgery after more than one year. Lastly, the lack of an upper age limit for age inclusion may have (slightly) skewed the results, based on life expectancy and working situation.

## Conclusion

This study showed that operative treatment of a humeral shaft fracture is more expensive than nonoperative treatment, but results in a higher health-related quality of life and significantly less disability. Due to the limited effect of a humeral shaft fracture on quality of life measured with the EQ-5D, the cost-effectiveness of operative treatment in terms of costs per QALY (€111,857) exceeds the acceptability limit. However, the incremental costs of €2880 per clinically meaningful difference in DASH are much lower and suggest that operative treatment for a humeral shaft fracture is cost-effective.

## Supplementary Information

Below is the link to the electronic supplementary material.

Supplementary file1 (DOCX 28 KB)

Supplementary file2 (DOCX 21 KB)

## Data Availability

No additional data are available. Data can be made available upon reasonable request to the principal investigator.
