# Publisher Correction: Economic evaluation of operative versus nonoperative treatment of a humeral shaft fracture: economic analyses alongside a multicenter prospective cohort study (HUMMER)

**DOI:** 10.1007/s00068-022-02210-8

**Published:** 2023-02-06

**Authors:** Saskia H. Van Bergen, Esther M. M. Van Lieshout, Kiran C. Mahabier, Alexandra J. L. M. Geraerds, Suzanne Polinder, Dennis Den Hartog, Michael H. J. Verhofstad, the HUMMER Investigators, Ivo Beetz, Hugo W. Bolhuis, P. Koen Bos, Maarten W. G. A. Bronkhorst, Milko M. M. Bruijninckx, Jeroen De Haan, Axel R. Deenik, P. Ted Den Hoed, Martin G. Eversdijk, J. Carel Goslings, Robert Haverlag, Martin J. Heetveld, Albertus J. H. Kerver, Karel A. Kolkman, Peter A. Leenhouts, Sven A. G. Meylaerts, Ron Onstenk, Martijn Poeze, Rudolf W. Poolman, Bas J. Punt, Ewan D. Ritchie, W. Herbert Roerdink, Gert R. Roukema, Jan Bernard Sintenie, Nicolaj M. R. Soesman, Edgar J. T. Ten Holder, Wim E. Tuinebreijer, Maarten Van der Elst, Frank H. W. M. Van der Heijden, Frits M. Van der Linden, Peer Van der Zwaal, Jan P. Van Dijk, Hans-Peter W. Van Jonbergen, Egbert J. M. M. Verleisdonk, Jos P. A. M. Vroemen, Marco Waleboer, Philippe Wittich, Wietse P. Zuidema, Ahmed Al Khanim, Jelle E. Bousema, Kevin Cheng, Yordy Claes, J. Daniël Cnossen, Emmelie N. Dekker, Aron J. M. De Zwart, Priscilla A. Jawahier, Boudijn S. H. Joling, Cornelia A. W. Notenboom, Jaap B. Schulte, Nina Theyskens, Gijs J. J. Van Aert, Boyd C. P. Van der Schaaf, Tim Van der Torre, Joyce Van Veldhuizen, Lois M. M. Verhagen, Maarten Verwer, Joris Vollbrandt

**Affiliations:** 1 Trauma Research Unit, Department of Surgery, Erasmus MC, University Medical Center Rotterdam, Rotterdam, The Netherlands; 2 Department of Public Health, Erasmus MC, University Medical Center Rotterdam, Rotterdam, The Netherlands

**Publisher Correction: European Journal of Trauma and Emergency Surgery** 10.1007/s00068-022-02160-1

In this article, the order that the authors appeared in the author list was incorrect.

The correct order is: Saskia H. Van Bergen1 · Esther M. M. Van Lieshout1 · Kiran C. Mahabier1 · Alexandra J. L. M. Geraerds2 · Suzanne Polinder2 · Dennis Den Hartog1 · Michael H. J. Verhofstad1 · on behalf of the HUMMER Investigators.

The original article has been corrected.

