# Supplementary material for: Economic evaluation of operative versus nonoperative treatment of a humeral shaft fracture: economic analyses alongside a multicenter prospective cohort study (HUMMER)
Source: Eur J Trauma Emerg Surg. 2022 Dec 8;49(2):929–38. doi: 10.1007/s00068-022-02160-1 (PMC10175317; doi:10.1007/s00068-022-02160-1)
Supplement: Supplementary file 1 — Supplementary file1 (DOCX 28 KB) [file 68_2022_2160_MOESM1_ESM.docx]

**Supplemental Table S1. Sources and unit costs of health care resources in 2020 (€)**

| **Cost category** | **Unit** | **Source of consumption data** | **Source of value** | **Unit price (**€) |
| --- | --- | --- | --- | --- |
| **Hospital costs – primary stay** |  |  |  |  |
| Ambulance transport | Ride | Hospital registry | NZa^1^ | 730 |
| Emergency department visit | Visit | Hospital registry | Cost manual^2^ | 280 |
| Medical imaging |  |  |  |  |
| Laboratory tests | Test | Hospital registry | NZa^1^ | 22^a^/28^b^ |
| X-ray upper extremity | X-ray | Hospital registry | NZa^1^ | 59 |
| CT-scan upper extremity | CT-scan | Hospital registry | NZa^1^ | 157 |
| MRI scan upper extremity | MRI scan | Hospital registry | NZa^1^ | 232 |
| EMG | EMG | Hospital registry | Hospital/industry data^3^ | 160 |
| Ultrasound | Ultrasound | Hospital registry | NZa^1^ | 94 |
| Arthrography | Arthrogram | Hospital registry | Hospital/industry data^3^ | 134 |
| Hospital admission days | Day | Hospital registry | Cost manual^2^ | 694^a^/479^b^ |
| Medication* | Dose  per day | Hospital registry/  Patient questionnaire^§^ | CVZ^4^ | Variable |
| Operative treatment |  |  |  |  |
| Surgeon | Hour | Hospital registry | Cost manual^2^ | 85^a^/88^b^ |
| Operating room** | Hour | Hospital registry | Hospital/industry data^3^ | 600^a^/708^b^ |
| Equipment and implants | Unit | Hospital registry | Hospital/industry data^3^ | Variable |
| Depuy Synthes MultiLoc IMN*** |  |  |  | 478 |
| MultiLoc IMN endcap |  |  |  | 38 |
| MultiLoc IMN screw^c^ |  |  |  | 65 |
| MultiLoc IMN 4.5mm screw |  |  |  | 76 |
| MultiLoc IMN locking screw |  |  |  | 54 |
| T2 Stryker IMN**** |  |  |  | 302 |
| T2 Stryker endcap |  |  |  | 35 |
| T2 Stryker screw |  |  |  | 31 |
| Depuy Synthes Titanium Elastic Nail (TEN)*** |  |  |  | 97 |
| Depuy Synthes Expert Humeral Nail (EHN)*** |  |  |  | 357 |
| EHN endcap |  |  |  | 36 |
| EHN spiral blade |  |  |  | 301 |
| EHN screw |  |  |  | 46 |
| Stryker AxSOS humeral plate**** |  |  |  | 527 |
| Stryker AxSOS locking screw |  |  |  | 59 |
| Depuy Synthes Philos proximal humeral plate*** |  |  |  | 699 |
| Depuy Synthes femur plate*** |  |  |  | 507 |
| Depuy Synthes metaphyseal plate*** |  |  |  | 304 |
| Depuy Synthes distal extra-articular*** |  |  |  | 841 |
| Depuy Synthes screw^c^ |  |  |  | 29 |
| Depuy Synthes locking screw |  |  |  | 34 |
| Depuy Synthes cortex screw |  |  |  | 25 |
| Devices for immobilization | Unit | Hospital registry | Variable | Variable |
| Sling or collar and cuff |  |  | Mean of tracked prices^5^ | 8 |
| Splint |  |  | Mean of tracked prices^5^ | 33 |
| Brace |  |  | Hospital/industry data^3^ | 103 |
| Hanging cast |  |  | Hospital/industry data^3^ | 53 |
| Pressure bandage |  |  | Mean of tracked prices^5^ | 2 |
| Gilchrist |  |  | Mean of tracked prices^5^ | 28 |
| Tubigrip |  |  | Mean of tracked prices^5^ | 8 |
| Nonoperative treatment |  |  |  |  |
| Devices for immobilization |  |  |  | Variable |
|  |  |  |  |  |
| **Hospital costs – follow-up** |  |  |  |  |
| Medical imaging |  |  |  | Variable |
| Medication* |  |  |  | Variable |
| General practitioner | Visit | Patient questionnaire^§^ | Cost manual^2^ | 36 |
| Outpatient clinic visits | Visit | Hospital registry/  Patient questionnaire^§^ | Cost manual^2^ |  |
| Surgeon |  |  |  | 79 |
| Rehabilitation doctor |  |  |  | 98 |
| Neurologist |  |  |  | 107 |
| Anesthetist |  |  |  | 98 |
| Geriatrician |  |  |  | 98 |
| Rheumatologist |  |  |  | 98 |
| Wound care nurse |  |  |  | 34 |
| Plaster cast nurse |  |  |  | 34 |
| Adverse events |  |  |  |  |
| Emergency department visit |  |  |  | 280 |
| Hospital admission days |  |  |  | 694^a^/479^b^ |
| Medication* |  |  |  | Variable |
| Revision surgery |  |  |  |  |
| Surgeon |  |  |  | 85^a^/88^b^ |
| Operating room** |  |  |  | 600^a^/708^b^ |
| Equipment and implants |  |  |  | Variable |
| Implant removal | Operation | Hospital registry | Hospital/industry data^3^ | - ^a^/- ^b^ |
| Radial nerve grafting | Operation | Hospital registry | Hospital/industry data^3^ | 67^a^/191^b^ |
| Incision and drainage | Operation | Hospital registry | Hospital/industry data^3^ | 41^a^/191^b^ |
| **Costs related to rehabilitation /**  **changes in living situation** |  |  |  |  |
| Discharge disposition |  |  |  |  |
| Care hotel | Days | Patient questionnaire^§^ | Cost manual^2^ | 178 |
| Elderly home | Days | Patient questionnaire^§^ | Cost manual^2^ | 182 |
| Nursing home | Days | Patient questionnaire^§^ | Cost manual^2^ | 182 |
| Rehabilitation center | Days | Patient questionnaire^§^ | Cost manual^2^ | 497 |
| Home care (household support/nursing care) | Hours | Patient questionnaire^§^ | Cost manual^2^ | 79 |
| Rehabilitation therapy |  |  |  |  |
| Physical therapy | Session | Patient questionnaire^§^ | Cost manual^2^ | 36 |
| Other rehabilitation therapy |  |  |  |  |
| Edema therapy | Session | Patient questionnaire^§^ | Cost manual^2^ | 42 |
| Occupational therapy | Session | Patient questionnaire^§^ | Cost manual^2^ | 36 |
| Hand therapy | Session | Patient questionnaire^§^ | Cost manual^2^ | 37 |
| Chiropractic therapy | Session | Patient questionnaire^§^ | Cost manual^2^ | 58 |
| Massage therapy | Session | Patient questionnaire^§^ | Cost manual^2^ | 59 |
| Manual therapy | Session | Patient questionnaire^§^ | Cost manual^2^ | 46 |
| Acupuncture therapy | Session | Patient questionnaire^§^ | Cost manual^2^ | 88 |
|  |  |  |  |  |
| **Work absence** |  |  |  |  |
| age 18-20 | Hours | Patient questionnaire^§^ | CBS^6^ | 7 |
| age 20-25 | Hours | Patient questionnaire^§^ | CBS^6^ | 13 |
| age 25-30 | Hours | Patient questionnaire^§^ | CBS^6^ | 18 |
| age 30-35 | Hours | Patient questionnaire^§^ | CBS^6^ | 22 |
| age 35-40 | Hours | Patient questionnaire^§^ | CBS^6^ | 25 |
| age 40-45 | Hours | Patient questionnaire^§^ | CBS^6^ | 27 |
| age 45-50 | Hours | Patient questionnaire^§^ | CBS^6^ | 28 |
| age 50-55 | Hours | Patient questionnaire^§^ | CBS^6^ | 28 |
| age 55-60 | Hours | Patient questionnaire^§^ | CBS^6^ | 28 |
| age 60-65 | Hours | Patient questionnaire^§^ | CBS^6^ | 28 |
| age 65-68 | Hours | Patient questionnaire^§^ | CBS^6^ | 25 |

The exchange rate was: €1 = US$ 1.21 [31].

* Humeral shaft fracture related medication only (see Supplemental Table S2 for details).

** Including operating room personnel, anesthesia, surgical equipment and overhead costs.

*** Depuy Synthes (Companies, Raynham, Massachusetts, United States).

**** Stryker Corporation (Kalamazoo, Michigan, United States).

^§^ Patient questionnaire; Customized version.

^1^ NZa; Nederlandse Zorgautoriteit (Dutch Healthcare Authority) standard costs [24].

^2^ Cost manual; Manual on cost research, methods and standard costs in economic healthcare evaluations, version 2015 [28].

^3^ Hospital/industry data; costs were requested from one academic hospital, one non-academic hospital, and one surgical equipment and implant firm. Means were calculated and used as an estimation of the costs in all participating hospitals.

^4^ CVZ; Standard prices were used as described by the CVZ (Supplemental Table S2) [29].

^5^ Mean of tracked prices; costs of aids were presented online by home care firms. These costs were used as an estimation of the real costs in all participating patients.

^6^ CBS; Costs of work absence 2020 (€) were used as described by the CBS [33].

^a^ Academic hospital, ^b^ Non-academic hospital, ^c^ Average screw prices were used when screw specifics were not attained.

CBS, Centraal Bureau voor de Statistiek (Statistics Netherlands); CVZ, College voor Zorgverzekeringen (Health Care Insurance Board); CT, Computed tomography; EHN, Expert humeral nail; IMN, Intramedullary nail; MRI, Magnetic resonance imaging; NZa, Nederlandse Zorgautoriteit (Dutch Healthcare Authority); TEN, Titanium elastic nail.
