# Supplementary material for: Economic evaluation of operative versus nonoperative treatment of a humeral shaft fracture: economic analyses alongside a multicenter prospective cohort study (HUMMER)
Source: Eur J Trauma Emerg Surg. 2022 Dec 8;49(2):929–38. doi: 10.1007/s00068-022-02160-1 (PMC10175317; doi:10.1007/s00068-022-02160-1)
Supplement: Supplementary file 2 — Supplementary file2 (DOCX 21 KB) [file 68_2022_2160_MOESM2_ESM.docx]

**Supplemental Table S2. Medication prices in 2020 (€)**

| **Medication name** | **Dose** | **ATC code*** | **Unit price (€)** |
| --- | --- | --- | --- |
| Amoxicillin | 625 mg | J01CR02 | 0.65 |
| Aspirin | 80 mg | N02BA01 | 0.05 |
| Cefazolin | 1 g | J01DB04 | 2.90 |
| Ciprofloxacin | 500 mg | J01MA02 | 0.11 |
| Clindamycin | 300 mg | J01FF01 | 0.39 |
| Diclofenac/Voltaren | 50 mg | M01AB05 | 3.18 |
|  | 75 mg | M01AB05 | 4.77 |
|  | 100 mg | M01AB05 | 6.36 |
|  | 75 mg | M01AB55 | 0.50 |
| Etoricoxib | 60 mg | M01AH05 | 0.33 |
|  | 90 mg | M01AH05 | 0.27 |
| Fentanyl (transdermal) | 12 microg/hour | N02AB03 | 0.02 |
|  | 25 microg/hour | N02AB03 | 0.03 |
| Fraxiparine | 9,500 IE anti-Xa/ml, 0.3ml | B01AB06 | 1.84 |
| Ibuprofen | 200 mg | M01AE01 | 0.05 |
|  | 400 mg | M01AE01 | 0.10 |
|  | 600 mg | M01AE01 | 0.08 |
|  | 800 mg | M01AE01 | 0.13 |
| Morphine (solution) | 5 mg/ml | N02AA01 | 0.16 |
| Morphine (injection) | 10 mg/ml | N02AA01 | 11.30 |
| Morphine (tablet) | 10 mg | N02AA01 | 0.32 |
|  | 15 mg | N02AA01 | 0.38 |
| Naproxen | 250 mg | M01AE02 | 0.07 |
|  | 500 mg | M01AE02 | 0.11 |
| Omeprazole | 10 mg | A02BC01 | 0.06 |
|  | 20 mg | A02BC01 | 0.05 |
|  | 40 mg | A02BC01 | 0.06 |
| Oxycodone | 5 mg | N02AA05 | 0.12 |
| Oxycontin | 5 mg | N02AA05 | 0.22 |
|  | 10 mg | N02AA05 | 0.33 |
| Oxynorm | 5 mg | N02AA05 | 0.45 |
|  | 10 mg | N02AA05 | 0.76 |
|  | 20 mg | N02AA05 | 0.60 |
|  | 30 mg | N02AA05 | 2.16 |
| Pantoprazole | 20 mg | A02BC02 | 0.05 |
|  | 40 mg | A02BC02 | 0.08 |
| Paracetamol | 500 mg | N02BE01 | 0.03 |
|  | 1 g | N02BE01 | 0.09 |
| Paracetamol/codeine | 500 mg/10 mg | N02BE51 | 0.05 |
|  | 500 mg/20 mg | N02BE51 | 0.07 |
| Piritramide | 10 mg/ml, 2 ml | N02AC03 | 1.85 |
| Tramadol | 50 mg | N02AX02 | 0.05 |
| Triamcinolonacetonide | 10 mg/ml | H02AB08 | 1.68 |
| Paracetamol, tramadol/Zaldiar | 325 mg/37.5 mg | N02AJ13 | 0.17 |

The exchange rate was: €1 = US$1.21 [31].

Standard prices were used as described by the CVZ (College voor Zorgverzekeringen; Health Care Insurance Board), online available on [www.medicijnkosten.nl](http://www.medicijnkosten.nl) [29].

ATC code; Anatomical Therapeutic Chemical Classification System; CVZ, College voor Zorgverzekeringen (Health Care Insurance Board).
